# Fisetin, an Anti-Inflammatory Agent, Overcomes Radioresistance by Activating the PERK-ATF4-CHOP Axis in Liver Cancer

**DOI:** 10.3390/ijms24109076

**Published:** 2023-05-22

**Authors:** Tae Woo Kim

**Affiliations:** Department of Biopharmaceutical Engineering, Dongguk University-WISE, 123 Dongdae-ro, Gyeongju 38066, Gyeongbuk, Republic of Korea; tae1410@naver.com or tae1410@dongguk.ac.kr

**Keywords:** liver cancer, fisetin, radiation, ER stress, cell death

## Abstract

Fisetin, a well-known plant flavonol from the natural flavonoid group, is found in traditional medicines, plants, vegetables, and fruits. Fisetin also has anti-oxidant, anti-inflammatory, and anti-tumor effects. This study investigated the anti-inflammatory effects of fisetin in LPS-induced Raw264.7 cells and found that fisetin reduced the LPS-induced production of pro-inflammation markers, such as TNF-α, IL-1β, and IL-6, demonstrating the anti-inflammatory effects of fisetin. Furthermore, this study investigated the anti-cancer effects of fisetin and found that fisetin induced apoptotic cell death and ER stress through intracellular calcium (Ca^2+^) release, the PERK-ATF4-CHOP signaling pathway, and induction of GRP78 exosomes. However, the suppression of PERK and CHOP inhibited the fisetin-induced cell death and ER stress. Interestingly, fisetin induced apoptotic cell death and ER stress and inhibited the epithelial-mesenchymal transition phenomenon under radiation in radiation-resistant liver cancer cells. These findings indicate that the fisetin-induced ER stress can overcome radioresistance and induce cell death in liver cancer cells following radiation. Therefore, the anti-inflammatory agent fisetin, in combination with radiation, may be a powerful immunotherapy strategy to overcome resistance in an inflammatory tumor microenvironment.

## 1. Introduction

Liver cancer has an overwhelming cancer death rate worldwide [1]. Liver cancer is often divided into several types, including primary liver cancers and secondary liver cancers [2]. Primary liver cancers such as sarcoma, cholangiocarcinoma, and hepatocellular carcinoma account for about 6% of all cancer deaths worldwide and derive from the liver [3]. Secondary liver cancers are derived from other organs, including breast, ovarian, pancreatic, and lung, and spread to the liver via the development of metastases [4].

Chemo-resistance is a well-known phenomenon that arises from various elements, including epigenetic changes, molecular mechanisms, and genetic mutations [5]. Radiation therapy using radiation exposure machines, including X-rays, alpha (α), beta (β), and gamma (γ)-rays, is a potential tumor therapy strategy; however, many patients experience therapeutic obstacles caused by radioresistance problems. In addition, although radiation exposure therapies can be effective in various cancers, including cervical, skin, prostate, non-small-cell lung, and head and neck carcinoma, they have a lower effect against other cancers, including bladder, breast, liver, and glioblastoma [6]. Radiotherapy and anti-cancer drugs can kill normal cells through toxic adverse effects, whereas herbal medicine may be a promising tumor therapeutic strategy to lessen the side effects.

Fisetin (3,7,3′,4′-tetrahydroxyflavone) is a naturally occurring dietary polyphenolic flavonol and is known to induce wide and potential bioactivities, such as antioxidant, anti-cancer, and anti-inflammatory effects in diverse cancer types [7,8,9]. Inflammation contributes to the development of cancer and promotes tumorigenesis and chemoresistance [10,11]. Anti-inflammatory agents frequently regulate immune mechanisms by inducing apoptosis in cancer cells and chemoresistance models [12]. Fisetin, an anti-inflammatory reagent, regulated caspase-3-mediated apoptotic cell death in mouse leukemia cells by inducing cell cycle arrest [13]. In human osteosarcoma cells, fisetin caused apoptosis by activating the hippo pathway and the JNK-ERK-AP-1 axis [14]. Fisetin has been shown to have anti-cancer effects against various cancer types, including lung, breast, renal, and gastric [15,16,17,18]. Flavonoids, such as apigenin and luteolin, exert anti-tumor effects through the activation of the endoplasmic reticulum (ER) stress response and the unfolded protein response (UPR) in various cancers [19,20]. The ER is important for various cellular activities, such as translation, synthesis, maturation, protein folding, and the sequestration of calcium (Ca^2+^) [21]. In the ER, problems with Ca^2+^ concentrations interrupt protein folding and lead to ER stress through the accumulation of unfolded proteins [22]. Ca^2+^ plays powerful roles in various cell signaling pathways, including growth factors, hormones, reactive oxygen species (ROS), and apoptosis [23,24]. ER stress also leads to the release of intracellular Ca^2+^, causing continuous and excessive ER stress, which induces apoptotic cell death in various cell types [25]. Many studies have suggested that fisetin might have a powerful anti-cancer effect in various cancers. However, studies on the anti-cancer effect and molecular mechanism of fisetin in liver cancer have not been reported. The ER stress-related signaling pathway has been reported to be one of the apoptotic cell death signaling pathways via the consequences of unfolded and misfolded proteins in several cancers [26]. UPR primary transducers, such as inositol-requiring-1 (IRE1), activating transcription factor-6 (ATF6), and pancreatic ER kinase (PERK), are in the ER membrane and activate the ER stress signaling pathway and protein folding [27]. Various stimuli initiate the ER stress response and cause PERK to phosphorylate and activate eukaryotic translation initiation factor-2α (eIF2α). Upon activation, eIF2α mediates the translation and translocation of activating transcription factor-4 (ATF4) and CCAAT-enhancer-binding protein homologous protein (CHOP) [28]. After this signaling activation, each cell signaling mechanism modulates downstream responses such as multiple cell death [29]. Flavonoids are natural antioxidants and exert anti-cancer functions in various cancers, and these frequently induce apoptosis and cell death via the ROS-dependent ER stress signaling pathway [30]. Fisetin activates apoptosis, cell death, and ER stress response through ROS and intracellular Ca^2+^ production in cancer cells [31,32].

This study aimed to investigate the underlying mechanisms to overcome radioresistance by inducing the GRP78-PERK-ATF4-CHOP pathway in fisetin-treated radioresistant liver cancer cells.

## 2. Results

### 2.1. Fisetin Decreases the Expression and Release of Pro-Inflammatory Cytokines in LPS-Mediated Raw264.7 Cells

**Figure 1:**
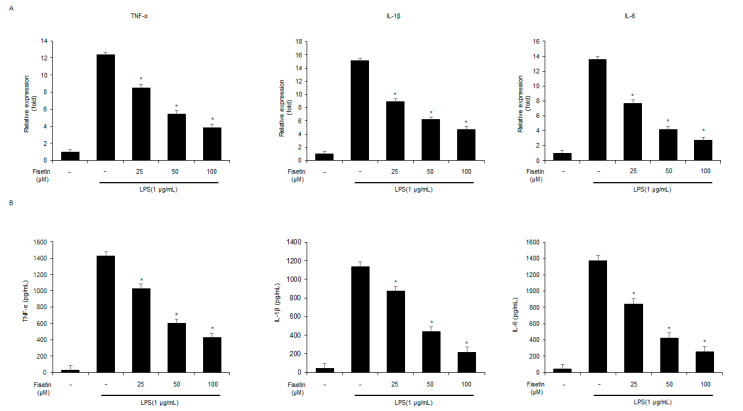
Fisetin decreases the inflammatory responses in LPS-mediated Raw264.7 cells. (**A**) Raw264.7 cells were pretreated with LPS (1 µg/mL) for 24 h and then treated with fisetin (25, 50, and 100 µM, 24 h). The expression of IL-6, IL-1β, and TNF-α were analyzed by RT-qPCR. Relative mRNA expression levels were normalized to β-actin. (**B**) The secretion levels of IL-6, IL-1β, and TNF-α were measured and analyzed by ELISA. * = *p* < 0.05. Data are representative of three experiments.

To identify the anti-inflammatory effects of fisetin on Raw264.7 cells, the mRNA levels of the pro-inflammatory cytokines IL-6, IL-1β, and TNF-α were investigated. Real-time PCR indicated that the expressions of TNF-α, IL-1β, and IL-6 were up-regulated in LPS-treated Raw264.7 cells. However, the expressions of IL-6, IL-1β, and TNF-α were down-regulated by fisetin in a dose-dependent manner in the LPS-treated Raw264.7 cells (Figure 1A). To identify if fisetin regulates the levels of IL-6, IL-1β, and TNF-α in LPS-treated Raw264.7 cells, an ELISA assay was performed. Fisetin significantly decreased the release of IL-6, IL-1β, and TNF-α in a dose-dependent manner in the medium of LPS-treated Raw264.7 cells (Figure 1B). These results indicate that in LPS-treated Raw264.7 cells, fisetin decreases with a dose-dependent pattern the expression and release of LPS-treated pro-inflammatory cytokines.

### 2.2. Fisetin Induces Apoptotic Cell Death in Liver Cancer Cells

**Figure 2:**
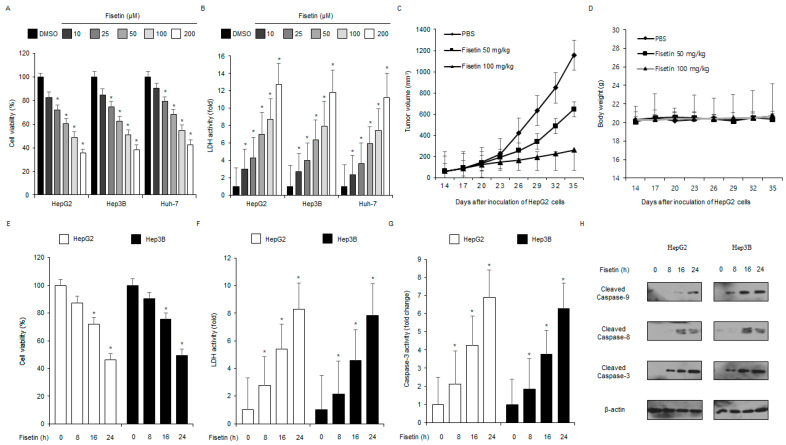
Anti-liver cancer effect of fisetin in vitro and in vivo. (**A**,**B**) LDH activity and cell viability after different fisetin treatments (10, 25, 50, 100, and 200 µM, 24 h) in liver cancer cells (HepG2, Hep3B, and Huh-7) were measured and analyzed using LDH activity and WST-1 assays. The cell viability of the DMSO-treated cells was set at 100%. * = *p* < 0.05. Data are representative of three experiments. (**C**,**D**) Xenograft nude mice were implanted (sc) with 1 × 10^7^ HepG2 cells into the right hind thigh and were randomly divided into the treatment groups (two groups, 50 or 100 mg/kg) and the control groups (*n* = 10/group). Fisetin (50 or 100 mg/kg) or PBS was administered (ip) once a day for two days. The body weights of the HepG2 tumor-xenograft mice were determined twice a week during the experiment. (**E**–**H**) The caspase-3 activity, cell cytotoxicity, and cell viability of the HepG2 and Hep3B cells treated with fisetin (100 µM) at different times (8, 16, and 24 h) were performed and analyzed using caspase-3 activity, LDH, and WST-1 assays. * = *p* < 0.05. Data are representative of three experiments. Western blotting for caspase-9, caspase-8, and caspase-3 cleavage was performed at the indicated times using the fisetin-treated HepG2 and Hep3B cells. Data are representative of three experiments.

To identify the cytotoxic effect of fisetin on various cancer cell types, cell viability and lactate dehydrogenase (LDH) assays were performed. As shown in Figure 2A,B, fisetin decreased the cell viability and increased LDH activity in HepG2, Hep3B, and Huh7 cells in a concentration-dependent manner (10, 25, 100, and 200 μM, 24 h). To further identify the anti-cancer effects of fisetin in vivo, a liver cancer xenograft mouse model was constructed with HepG2 cells. Mice in the 50 mg/kg and 100 mg/kg fisetin groups were monitored and had lower tumor volumes than the control group in a dose-dependent manner (Figure 2C). Body weights did not significantly differ among the groups (Figure 2D). To determine the time course of the cytotoxic effect of fisetin in the liver cancer cell lines HepG2 and Hep3B, WST-1, caspase-3, and LDH activity assays were performed at the indicated times (0, 8, 16, and 24 h, 100 μM). As shown in Figure 2E–G, the cell viability was decreased, and the LDH and caspase-3 activity was significantly enhanced in a time-dependent (100 μM; 0, 8, 16, and 24 h) fisetin-treated HepG2 and Hep3B cells. To better investigate the link between fisetin treatment and caspase-dependent apoptosis, western blot analyses were performed. The fisetin treatment significantly increased the pro-apoptotic markers, including cleaved caspase-3, caspase-8, and caspase-9 over indicated time points (Figure 2H).

**Figure 3:**
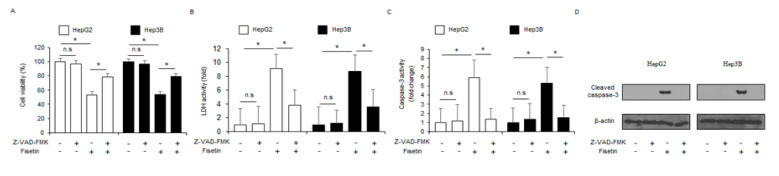
Fisetin, an anti-inflammatory agent, induces apoptosis in a caspase-dependent manner in liver cancer cells. (**A**–**D**) The effect of the pan-caspase inhibitor Z-VAD-FMK (50 mM, 24 h) on fisetin-treated apoptotic cell death. HepG2 and Hep3B cells were pretreated with Z-VAD-FMK (50 mM, 4 h) and then treated with fisetin (100 µM, 24 h). Caspase-3 activity, LDH, and WST-1 assays were carried out. * = *p* < 0.05, n.s = no significant. The protein expression level of cleaved caspase-3 was determined and analyzed by western blotting. The level of β-actin was used as the protein loading control. Data are representative of three experiments.

To confirm whether fisetin is modulated by a pan-caspase inhibitor (Z-VAD-FMK), HepG2 and Hep3B cells were treated with fisetin (100 μM, 24 h) and Z-VAD-FMK (50 µM, 24 h). Results show that Z-VAD-FMK inhibited the reduction of cell viability and decreased caspase-3 and LDH activity in fisetin-treated liver cancer cells (Figure 3A–C). The western blotting demonstrated that fisetin, in combination with Z-VAD-FMK, decreased fisetin-induced caspase-3 cleavage (Figure 3D).

### 2.3. Fisetin Induces Apoptosis and Cell Death via ER Stress Signaling in Liver Cancer Cells

**Figure 4:**
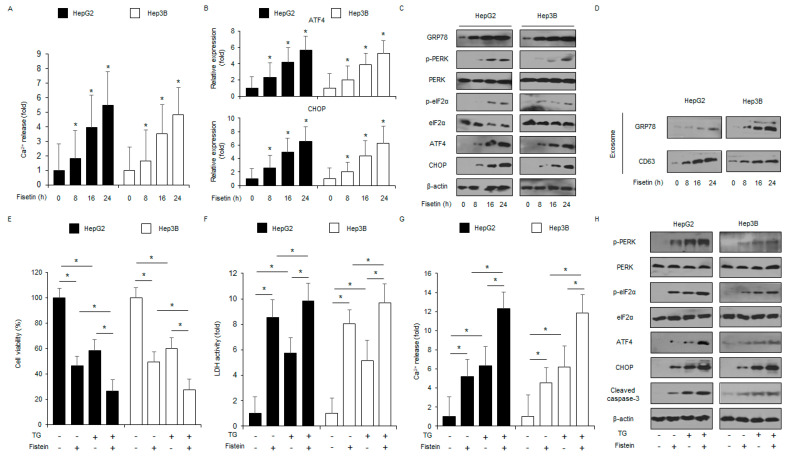
Fisetin induces ER stress in liver cancer cells. (**A**) HepG2 and Hep3B cells were treated with fisetin (100 μM) for the indicated times, and an intracellular Ca^2+^ assay was performed. * = *p* < 0.05. (**B**) The ER stress markers ATF4 and CHOP were measured by RT-PCR. Fold changes to target genes were normalized to β-actin. (**C**) The ER stress markers CHOP, PERK, eIF2α, p-eIF2α, p-PERK, ATF4, and GRP78 were measured by a western blot assay. β-actin was used as the protein loading control. (**D**) HepG2 and Hep3B cells were treated with fisetin (100 μM) for the indicated times, and then the exosomes (30 μg) were collected from the cell supernatant. Total exosomes were determined by western blotting using the exosome marker CD63 and the ER stress marker GRP78. (**E**–**H**) Cell viability, LDH activity, caspase-3 activity, intracellular Ca^2+^, and ER stress-related protein (cleaved caspase-3, p-eIF2α, ATF4, PERK, CHOP, eIF2α, and p-PERK) levels were measured in the thapsigargin (TG; 3 μM, 24 h) and fisetin (100 μM, 24 h)-treated HepG2 and Hep3B cells. * = *p* < 0.05. Data are representative of three experiments.

Many reports have indicated that ER stress plays an essential role in apoptotic cell death [33]. To evaluate if fisetin modulates the ER stress-related signaling pathway in liver cancer cells, we used. The intracellular Ca^2+^ release assay suggests that fisetin mediated the intracellular Ca^2+^ release in a time-dependent manner (Figure 4A). To confirm the mRNA levels of ER stress markers such as ATF4 and CHOP, Real-time PCR was performed. The fisetin treatment of HepG2 and Hep3B cells resulted in the upregulation of ATF4 and CHOP in a time-dependent manner (Figure 4B). To check the levels of ER stress-related proteins, such as PERK, eIF2α, CHOP, p-PERK, GRP78, p-eIF2α, and ATF4 in a time-dependent manner, western blot analyses were carried out. The fisetin treatment increased CHOP, p-eIF2α, ATF4, p-PERK, and GRP78 levels (Figure 4C). Emerging reports have suggested that GRP78 is released into the extracellular space through exosomes in cancer cells [34]. To analyze the role of GRP78 on fisetin-induced exosome release, HepG2 and Hep3B cells were treated with fisetin, and the exosomes secreted were purified from the cell culture supernatants. The fisetin treatment enhanced the secretion of the exosome marker CD63 in a time-dependent manner, and GRP78 expression was notably higher in the exosomes derived from the fisetin-treated cell culture supernatants compared with the control (Figure 4D). These findings suggest that exosomal GRP78 might be relevant to fisetin-induced ER stress and apoptotic cell death. The effect of the ER stress inducer thapsigargin (TG) in combination with fisetin on liver cancer cells was checked. Combination experiments indicated that fisetin combined with TG reduced cell viability and enhanced Ca^2+^ release and LDH activity compared with the control (Figure 4E–G). In addition, fisetin, in combination with TG, increased CHOP, p-eIF2α, p-PERK, cleaved caspase-3, ATF4, and GRP78 levels (Figure 4H).

### 2.4. Targeting the ER Stress Signaling Pathway Blocks Apoptotic Cell Death via Intracellular Ca^2+^ Production in Fisetin-Treated Liver Cancer Cells

**Figure 5:**
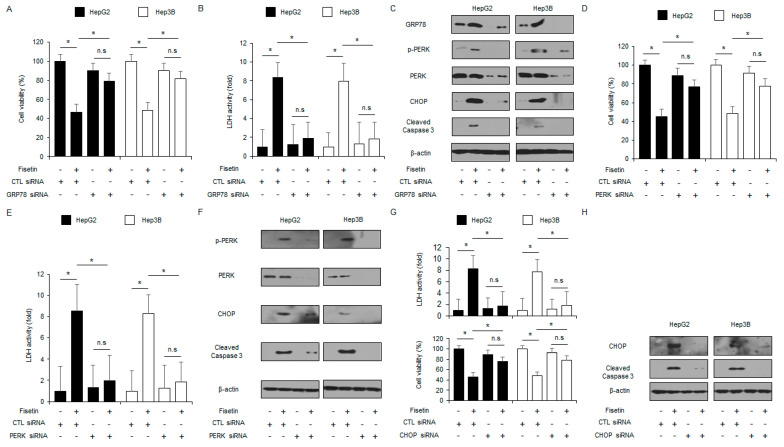
Targeting ER stress inhibits fisetin-induced apoptotic cell death in liver cancer cells. (**A**–**C**) HepG2 and Hep3B cells were transfected with GRP78 siRNA and treated with fisetin (100 μM, 24 h). LDH activity, Cell viability, and ER stress-related protein (CHOP, PERK, p-PERK, GRP78, and cleaved caspase-3) levels were measured. * = *p* < 0.05, n.s = no significant. Relative mRNA expression levels were normalized to β-actin. β-actin was used as the protein loading control. (**D**–**F**) Cell viability, LDH activity, and ER stress-related protein (cleaved caspase-3, CHOP, PERK, and p-PERK) levels in the HepG2 and Hep3B cells treated with fisetin (100 μM, 24 h) were measured in the presence or absence of PERK siRNA (30 nM, 24 h). * = *p* < 0.05. β-actin was used as the protein loading control. (**G**,**H**) LDH activity, Cell viability, and western blot analyses for CHOP and cleaved caspase-3 in HepG2 and Hep3B cells treated with fisetin (100 μM, 24 h) were performed in the presence or absence of CHOP siRNA (30 nM, 24 h). * = *p* < 0.05. β-actin was used as the protein loading control. Data are representative of three experiments.

To identify if GRP78 modulates apoptotic cell death in fisetin-treated liver cancer cells, GRP78 knockdown was studied. Targeting GRP78 using GRP78-specific siRNA increased cell viability and reduced LDH activity in fisetin-treated HepG2 and Hep3B cells compared with the control cells (Figure 5A,B). Compared to the control, the fisetin treatment induced the suppression of CHOP, p-PERK, cleaved caspase-3, and GRP78 expression in the GRP78 knockdown HepG2 and Hep3B cells. (Figure 5C). The PERK signaling pathway is a critical element in overcoming chemoresistance in cancer therapy [35]. A loss of function study was done by using PERK siRNA in fisetin-treated liver cancer cells. HepG2 and Hep3B cells were transfected with PERK siRNA (30 nM, 24 h) and then treated with fisetin. In the control cells, fisetin reduced cell viability and increased LDH activity, whereas, in the PERK knockdown cells, fisetin decreased LDH activity and increased cell viability (Figure 5D,E). The western blot analyses showed that the fisetin treatment enhanced the levels of ATF4, cleaved caspase-3, p-PERK, PERK, CHOP, and p-eIF2α compared with the control cells, whereas the PERK knockdown cells suppressed the levels of cleaved caspase-3, p-PERK, and CHOP with or without the fisetin treatment (Figure 5F). In the CHOP loss-of-function study, the fisetin treatment decreased cell viability and increased LDH activity in the control cells, whereas the fisetin-treated CHOP-impaired cells had less LDH activity and higher cell viability than the control cells (Figure 5G). The western blot analyses showed that the control cells had increased levels of cleaved caspase-3 and CHOP with the fisetin treatment, whereas the CHOP-impaired cells suppressed the levels of cleaved caspase-3 and CHOP with the fisetin treatment (Figure 5H).

### 2.5. Radiation Combined with Fisetin Overcomes Radioresistance via the Inhibition of the EMT Phenomenon in Radioresistant Liver Cancer Cells

**Figure 6:**
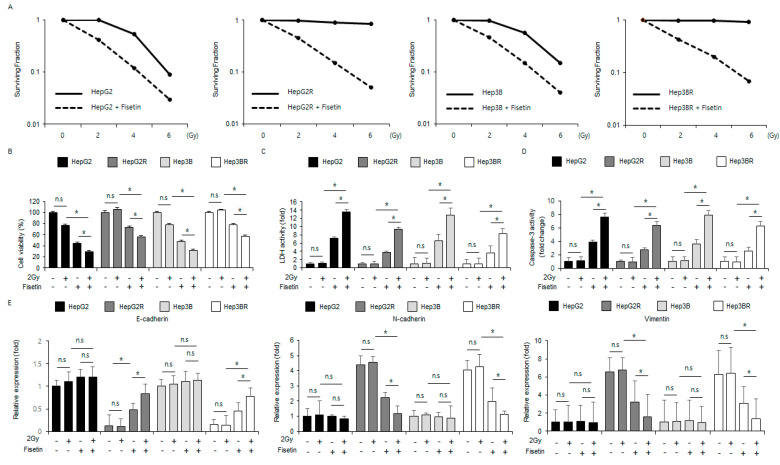
Fisetin, in combination with radiation (2 Gy), inhibits the EMT phenotype in radio-resistant liver cancer cells. (**A**) A colony assay was performed on HepG2, Hep3B, HepG2R, and Hep3BR cells treated with fisetin (100 μΜ, 24 h) after exposure to the indicated radiation doses (0, 2, 4, or 6 Gy). The survival fraction was analyzed using the surviving fraction formula. * = *p* < 0.05. (**B**–**D**) HepG2, Hep3B, HepG2R, and Hep3BR cells were treated with fisetin (100 μΜ, 24 h) after radiation exposure (2 Gy). LDH activity, Cell viability, and caspase-3 activity were determined using LDH, WST-1, and caspase-3 activity assays. (**E**) E-cadherin, N-cadherin, and vimentin mRNA levels were measured using RT-PCR. * = *p* < 0.05, n.s = no significant. β-actin was used as the protein loading control. Data are representative of three experiments.

To investigate whether fisetin overcomes radioresistance in cancer therapies, western blot analyses, cell viability assays, and colony formation assays were carried out in HepG2 and Hep3B cells and radio-resistant HepG2 (HepG2R) and Hep3B (Hep3BR) cells. When compared with the control cells, the fisetin treatment lowered the surviving fraction levels at different radiation exposures (2, 4, or 6 Gy) in HepG2, HepG2R, Hep3B, and Hep3BR cells (Figure 6A). In the HepG2 and Hep3B cells, fisetin reduced cell viability and enhanced caspase-3 and LDH activity, radiation (2 Gy) combined with fisetin reduced cell viability and increased caspase-3 activity and LDH activity even further, and radiation (2 Gy) alone had no effect (Figure 6B–D). In the HepG2R and Hep3BR cells, the fisetin treatment reduced cell viability and enhanced caspase-3 activity and LDH activity, radiation (2 Gy) combined with fisetin reduced cell viability and enhanced caspase-3 activity and LDH activity even further and radiation (2 Gy) alone had no effect (Figure 6B–D). To identify whether fisetin in combination with radiation regulates the epithelial-mesenchymal transition (EMT) phenomenon in radio-resistant liver cells, HepG2, HepG2R, Hep3B, and Hep3BR cells were treated with radiation (2 Gy) and fisetin. Real-time PCR analysis indicated that fisetin and fisetin combined with radiation (2 Gy) reduced the vimentin and N-cadherin expression and up-regulated E-cadherin in HepG2R and Hep3BR cells, whereas the expression of vimentin, N-cadherin, and E-cadherin was largely unchanged in the HepG2 and Hep3B cells (Figure 6E). In addition, when these cells were treated with fisetin and radiation (2 Gy), the expression of vimentin and N-cadherin were decreased, and the expression of E-cadherin was increased in the HepG2R and Hep3BR cells (Figure 6E). These findings suggested that fisetin combined with radiation (2 Gy) could help to overcome radioresistance by suppressing the EMT phenomenon in HepG2R and Hep3BR cells.

## 3. Discussion

Fisetin has various pharmacological properties, such as anti-inflammatory, anti-tumor, and anti-allergy effects. This study found that fisetin mediates apoptotic cell death by activating the ER stress signaling pathway in liver cancer cells. Fisetin treatments decreased the growth of liver cancer cells in vitro and in vivo. Fisetin, in combination with radiation, showed anti-proliferative effects in liver cancer cells and overcame radioresistance through the activation of the PERK signaling pathway and the inhibition of the EMT phenomenon in radio-resistant liver cancer cells.

Even though radiotherapy is powerful and is frequently used as a therapeutic approach for cancer patients, it still has serious side effects and problems, including a decrease in quality of life, safety concerns, resistance, and pain [36,37,38]. Bioactive compounds extracted from plants can help to reduce serious radiotherapy-induced side effects and problems. Combination therapies of radiation and herbal medicine have shown synergistic anti-cancer effects [39]. Radio- and chemo-therapies frequently cause hepatic injuries and toxicity in liver cancer patients, whereas the antioxidant extracts lycopene and ashwagandha, extracted from plants, significantly decreased irradiation-induced liver injuries and toxicity [40,41]. Recent reports have suggested that herbal medicines exert powerful anti-cancer effects by reducing the side effects of radiotherapy [42]. In addition, the combination therapy of natural medicine and radiation is considered to have synergistic effects by inhibiting radioresistance and removing cancer cells [43]. Natural sources, such as crude flavonoids, phenolic compounds, alkaloids, lectins, terpenoids, and flavors extracted from plants, may be potential radiosensitizers during radiotherapy for cancer patients [44]. Herbal drugs, in combination with radiation, frequently suppress tumor growth, angiogenesis, invasion, and metastasis via apoptosis and cell death, including morphology changes, cell membrane shrinkage, DNA fragmentation, cell cycle arrest, decreased cytosol organelles, and diverse molecular mechanisms in cancer cells [45]. The synthetic flavone flavopiridol combined with radiation (5 Gy) induced apoptotic cell death by targeting p53 and Bcl-2 in various cancer cell types and radio-resistant cancer cells [46]. Methyl jasmonate combined with radiation (7.6 Gy) mediated apoptotic cell death via the suppression of Bcl-2 and the activation of caspase-3 cleavage in human prostate adenocarcinoma cells (PC3) [47]. Caffeine combined with radiation (5 Gy) sensitized tumor cells and tissues by increasing cyclin B1 and caspase-3 cleavage expression, In vitro and in vivo, in LM3 hepatocellular carcinoma cells [48]. Although radiotherapy is a powerful anti-tumor therapeutic strategy, liver cancers frequently acquire radioresistance during radiation exposure [49,50]. The EMT phenomenon induced by radiation exposure causes the up-regulation of mesenchymal markers, such as N-cadherin and vimentin, and the inhibitory effects of the epithelial marker E-cadherin, which plays important roles in radioresistance [51,52]. Radioresistance caused by the EMT process induces poor prognosis and therapy failure by metastasis and cancer recurrence [53]. Combination therapies with radiation and anti-cancer drugs such as cisplatin, docetaxel, and etoposide might be anti-cancer therapeutic strategies to overcome radioresistance; however, combination therapies also have side effect problems [54,55]. To overcome radioresistance and side effects in cancer therapies, natural medicines in combination with radiation might be novel anti-cancer therapeutic strategies [56,57]. The O-methylated flavonol rhamnetin and the natural flavonoid cirsiliol overcame radioresistance via the downregulation of Notch1, Ciap1, Ciap2, survivin, vimentin, and fibronectin and the up-regulation of E-cadherin in radio-exposed non-small cell lung cancer cells [58]. Our finding indicated that fisetin, in combination with radiation, regulates the EMT phenomenon in radio-resistant liver cells, HepG2, HepG2R, Hep3B, and Hep3BR cells. Fisetin combined with radiation (2 Gy) reduced the vimentin and N-cadherin expression and up-regulated E-cadherin in HepG2R and Hep3BR cells. Recent reports have suggested that ER stress mediates apoptosis and overcomes radioresistance during radiation exposure [59]. Artocarpin extracted from the Artocarpus plant induced apoptotic cell death by activating ER stress and by producing ROS in human osteosarcoma cells [60]. In the present study, fisetin induces apoptotic cell death by activating intracellular Ca^2+^ release and ER stress signaling pathway (PERK-CHOP axis) in liver cancer cells.

The ER stress response acts as a potential factor in regulating tumor growth and invasion [61]. With prolonged and accumulated ER stress response, the activation of ER stress by the unfolded protein response (UPR) mediates apoptotic cell death in tumor cells [62]. This study’s findings suggest that fisetin mediates apoptosis and cell death via the activation of ER stress (PERK signaling pathway) in liver cancer cells. Exosomes extracted from the fisetin-treated liver cancer cells had an abundance of the exosomal ER stress marker GRP78. Furthermore, fisetin-treated intracellular Ca^2+^ release mediated the disturbance in ER function and the accumulation and consequence of unfolded proteins. This release also can be induced by the excessive and prolonged ER stress response, and then it mediates apoptotic cell death in liver cancer cells. The xenograft mice experiment indicated a dramatic decrease in tumor volume by the fisetin treatment after the inoculation of HepG2 cells. However, clinical trials could not be performed in this study. Clinical trials using fisetin might have risks and benefits. Recent clinical trials have reported that fisetin (100 mg) supplements taken for seven consecutive weeks significantly reduced inflammation markers such as IL-8 and hs-CRP in colorectal cancer patients and that fisetin may be a novel complementary anti-tumor agent [63]. Fisetin decreased the release and expression of IL-6, IL-1β, and TNF-α in LPS-treated Raw264.7 cells. Recent reports have indicated that the combination of natural compounds and radiation act as a powerful anti-cancer therapeutic strategy [64,65]. In the radioresistance liver cancer cell models, HepG2R and Hep3B, the combination treatment of fisetin and radiation overcame radioresistance and induced apoptotic cell death by downregulating N-cadherin and vimentin and upregulating E-cadherin. Therefore, fisetin is latently considerable for the therapy of liver cancer combined with radiation exposure.

In conclusion, this study suggests that fisetin suppresses tumor growth via apoptotic cell death and ER stress in liver cancer in vitro and in vivo. These results add to the scientific understanding of the anti-cancer mechanism of fisetin in liver cancer therapies. Moreover, this study made a new link between natural products and radiotherapy, which proposes a new approach to cancer treatments.

## 4. Materials and Methods

### 4.1. Reagents

Fisetin (F4043), thapsigargin (TG; ER stress inducer; T9033), lipopolysaccharide (LPS; L4391), and Z-VAD-FMK (a caspase inhibitor; V116) were purchased from Sigma-Aldrich (St. Louis, MO, USA).

### 4.2. Cell Culture

Human liver cancer cell lines (HepG2, Hep3B, and Huh-7) were purchased from the Korean Cell Line Bank (Cancer Research Center, Seoul National University, Seoul, Republic of Korea). These cell lines were cultured in DMEM, and RPMI1640 mediums (Welgene, Daegu, Republic of Korea) were added with 10% fetal bovine serum (FBS; Gibco Invitrogen, Adelaide, SA, Australia), penicillin (100 U/mL) and streptomycin (100 mg/mL; Welgene, Gyeongsan-si, Republic of Korea) at 37 °C with 5% CO_2_. The macrophage cell line Raw264.7 was obtained from the American Type Culture Collection (Rockville, MD, USA) and maintained in DMEM (Gibco, Grand Island, NY, USA) containing 10% FBS (HyClone, Logan, UT, USA) and antibiotics (100 U/mL penicillin and 100 μg/mL streptomycin; Gibco-BRL). These above cell lines were incubated at 37 °C in a 5% CO_2_ air incubator.

### 4.3. Cytokine Measurement

To measure cytokines from cell supernatants in fisetin-treated Raw264.7 cells, an enzyme-linked immunosorbent assay (ELISA) was performed. In the Raw264.7 cells, the levels of IL-6, IL-1β, and TNF-α were measured using a mouse IL-6 ELISA kit (DY-406; R&D Systems, Minneapolis, MN, USA), mouse IL-1β ELISA kit (DY-401; R&D Systems), and mouse TNF-α ELISA kit (DY-410; R&D Systems). All ELISAs were carried out following the manufacturer’s instructions.

### 4.4. Cell Viability Assay

Liver cancer cells (1 × 10^4^ cells/well) were seeded in a 96-well plate, and cell viability was measured and monitored using a WST-1 assay (Roche Applied Science, Indianapolis, IN, USA). The WST-1 assay was performed by adding 10 μL of the WST-1 reagent to each well of the 96-well plate, and then the plate was incubated in a humidified incubator with 5% CO_2_ at 37 °C. The absorbance of each well was measured at 450 nm using a microplate reader (Molecular Devices, San Jose, CA, USA). All cell viability assays were performed following the manufacturer’s instructions.

### 4.5. LDH Assay

Liver cancer cells (1 × 10^4^ cells/well) were seeded onto a 96-well plate with a growth medium. Cell cytotoxicity was analyzed and monitored with a Pierce LDH activity assay kit according to the manufacturer’s protocol (Thermo Scientific, Waltham, MA, USA). Briefly, 100 μL of the LDH reagent was added to each well of the 96-well plate, and then the plate was incubated in a dark room at room temperature. The absorbance of each well was measured at 490 or 492 nm using a microplate reader (Molecular Devices, CA, USA).

### 4.6. Caspase-3 Activity Assay

Liver cancer cells (1 × 10^4^ cells/well) were seeded into a 96-well plate with a growth medium. The caspase-3 activity was analyzed and monitored using a caspase-3 activity assay kit (colorimetric; Abcam, Milpitas, CA, USA) according to the manufacturer’s instructions. The absorbance of each well was measured at 405 nm using a microplate reader (Molecular Devices, CA, USA).

### 4.7. Ionizing Radiation (IR)

Different ionizing radiation (IR) exposure (2, 4, and 6 Gy) were performed using ^137^Cs as the radiation source (Atomic Energy of Canada, Ltd., Mississauga, ON, Canada).

### 4.8. Development of Acquired Radio-Resistant Liver Cancer Cell Lines

HepG2 and Hep3B cells were seeded and cultured to approximately 50–60% confluency and then exposed to 2 Gy of radiation daily for three months. Radio-resistant liver cancer cell lines (HepG2R and Hep3BR) were established from the respective parental cell lines (HepG2 and Hep3B).

### 4.9. Colony Formation Assay

Liver cancer cells (HepG2, HepG2R, Hep3B, and Hep3BR) were plated and incubated in 60 mm dishes at a density of 1000 cells/dish. After incubation, colonies were fixed and stained with 1% methylene blue in 50% ethanol. The survival fraction was calculated using the following formula: surviving fraction = the number of colonies formed/number of cells seeded × plating efficiency of the control group.

### 4.10. Transfection

Liver cancer cells (4 × 10^5^ cells/well) in a 6-well plate were transfected with double-stranded siRNAs (30 nmol/mL, Santa Cruz, Dallas, TX, USA) targeting CHOP (Bioneer; 1649-1, Daejeon, Republic of Korea), PERK (Santa Cruz; sc-36213), and GRP78 (Santa Cruz; sc-29338) for 24 h using Lipofectamine 2000 reagent (Invitrogen, Carlsbad, CA, USA) according to the manufacturer’s protocol.

### 4.11. Isolation of Total RNA

Total RNA from liver cancer cells (2 × 10^6^ cell/well) and Raw264.7 cells (2 × 10^6^ cell/well) cultured in 100-mm cell culture dishes was isolated using Trizol reagent according to the manufacturer’s protocols (Invitrogen, Carlsbad, CA, USA). Reverse transcription (cDNA) was generated using 10 µg of total RNA and a power cDNA synthesis kit (iNTRON, Seongnam, Republic of Korea).

### 4.12. Real-Time RT-PCR

PCR reactions were performed in triplicate for each sample using an ABI Power SYBR green PCR Master Mix (Applied Biosystems, Foster City, CA, USA) with primers specific for TNF-α 5′-ACGGCATGGATCTCAAAGAC-3′ (sense) and 5′-TGAGATAGCAAATCGGCTGAC-3′ (antisense), IL-1β 5′-GAGTGTGGATCCCAAGCAAT-3′ (sense) and 5′-CTTGTGCTCTGCTTGTGAGG-3′ (antisense), IL-6 5′-CTGATGCTGGTGACAACCAC-3′ (sense) and 5′-TCCACGATTTCCCAGAGAAC-3′ (antisense), ATF4 5′-AAGCCTAGGTCTCTTAGATG-3′ (sense) and 5′-TTCCAGGTCATCTATACCCA-3′ (antisense), CHOP 5′-ATGAGGACCTGCAAGAGGTCC-3′ (sense) and 5′-TCCTCCTCAGTCAGCCAAGC-3′ (antisense), E-cadherin 5′-GAACGCATTGCCACATACAC-3′ (sense) and 5′-GAATTCGGGCTTGTTGTCAT-3′ (antisense), N-cadherin 5′-GGCATACACCATGCCATCTT-3′ (sense) and 5′-GTGCATGAAGGACAGCCTCT-3′ (antisense), and vimentin 5′-CCAGGCAAAGCAGGAGTC-3′ (sense) and 5′-CGAAGGTGACGAGCCATT-3′ (antisense) using a Roche Light Cycler 96 System (Roche, Mannheim, Germany). The fold changes of target genes were normalized to β-actin (5′-AAGGCCAACCGCGAGAAGAT-3′ (sense) and 5′-TGATGACCTGGCCGTCAGG-3′ (antisense)), and the gene expression was quantified and analyzed using the 2^−ΔΔCt^ method.

### 4.13. Isolation of Protein

Protein cell lysates from liver cancer cells (2 × 10^6^ cells/well) were collected in RIPA buffer containing a protease inhibitor cocktail (Sigma-Aldrich, St. Louis, MO, USA). The supernatant was analyzed for protein content using the BCA method (Thermo Scientific, Carlsbad, CA, USA).

### 4.14. Western Blotting Analyses

Equal amounts of protein (20 μg) were size-fractionated by an 8–15% SDS-PAGE gel and then were transferred onto a nitrocellulose membrane (Millipore Corporation, Billerica, MA, USA). The following primary antibodies (1:1000) were used: eIF2α (Santa Cruz; sc-133132), β-actin (Santa Cruz; sc-47778), CD63 (Abcam, ab216130), cleaved caspase-9 (CellSignaling, #20750, Danvers, MA, USA), cleaved caspase-8 (CellSignaling, #9748), cleaved caspase-3 (CellSignaling, #9661), p-PERK (Thr980) (CellSignaling, #12185), ATF4 (CellSignaling, #11815), GRP78 (CellSignaling, #3177), CHOP (CellSignaling, #2895), PERK (CellSignaling, #5683), and p-eIF2α (Ser51; CellSignaling, #3398). The following secondary antibodies were used: rabbit anti-mouse IgG HRP-linked antibody (Santa Cruz, 1:6000, sc-358914) and mouse anti-rabbit IgG HRP-linked antibody (Santa Cruz, 1:6000, sc-2357). The blots were visualized using the D-Plus ECL Pico System (DonginLS, Seoul, Republic of Korea, ECL-PS100).

### 4.15. Exosomes Isolation

Liver cancer cells (2 × 10^6^ cells/well) were seeded into a 100 mm cell culture dish in a growth medium, and then exosomes were obtained from the cell culture supernatant from the fisetin (100 μM)-treated liver cancer cells according to the manufacturer’s protocol (Total Exosome Isolation Reagent for cell culture media, Thermo Scientific, Carlsbad, CA, USA).

### 4.16. Intracellular Ca^2+^ Assays

HepG2 and Hep3B cells were plated in a 96-well plate with a growth medium at 1 × 10^4^ cells/well. After 24 h, the cells were treated with fisetin for 24 h. An intracellular Ca^2+^ activity assay (Abcam, Ca^2+^ Assay Kit [Colorimetric]) was performed and analyzed as described in the supplier’s manual (Abcam, Cambridge, MA, USA).

### 4.17. Animals

For the animal study, 5-week-old female, athymic BALB/c nude mice (*nu*/*nu*) were purchased from OrientBio, Inc. (Daejeon, Republic of Korea) and maintained for 1 week with free access to sterile standard mouse chow (NIH-7 open formula) and water. Mice were housed randomly at 50 ± 20% humidity and approximately 21 ± 2 °C on a 12-h light/dark cycle (*n* = 10 mice/group). All experimental animal procedures were performed according to the National Institutes of Health guidelines, and the protocol was approved by the Institutional Animal Care and Use Committee of Kyung Hee University (KHSASP-20-250).

### 4.18. Tumor Xenograft Mouse Models

For the mice xenograft experiment, 6-week-old mice were inoculated with HepG2 human liver cancer cells by subcutaneously (sc) implanting 1 × 10^7^ cells into the right thigh. Six days later, the mice were randomly grouped (*n* = 10 per group), and fisetin (50 or 100 mg/kg) was administered intraperitoneally (ip) every other day. Tumor sizes were measured on 2 axes (*L*, longest axis; *W*, shortest axis) 3 times per week using Vernier calipers. Tumor volume was calculated as follows: (*L* × *W*^2^)/2.

### 4.19. Statistical Analysis

Data are expressed as the mean ± standard error (SE). Statistical analyses of the experimental data were performed using a two-sided Student’s *t*-test. *p*-values less than 0.05 were determined to indicate statistical significance. Data are representative of three experiments.

## Data Availability

All original data and images are contained within the article.
